# Disease association and inter-connectivity analysis of human brain specific co-expressed functional modules

**DOI:** 10.1186/s40659-015-0061-4

**Published:** 2015-12-16

**Authors:** Kimin Oh, Taeho Hwang, Kihoon Cha, Gwan-Su Yi

**Affiliations:** Department of Bio and Brain Engineering, KAIST, Daejeon, Korea; Department of Information and Communications Engineering, KAIST, Daejeon, Korea

**Keywords:** Co-expressed functional module, Disease association, Inter-connectivity analysis

## Abstract

**Background:**

In the recent studies, it is suggested that the analysis of transcriptomic change of functional modules instead of individual genes would be more effective for system-wide identification of cellular functions. This could also provide a new possibility for the better understanding of difference between human and chimpanzee.

**Results:**

In this study, we analyzed to find molecular characteristics of human brain functions from the difference of transcriptome between human and chimpanzee’s brain using the functional module-centric co-expression analysis. We performed analysis of brain disease association and systems-level connectivity of species-specific co-expressed functional modules.

**Conclusions:**

Throughout the analyses, we found human-specific functional modules and significant overlap between their genes in known brain disease genes, suggesting that human brain disorder could be mediated by the perturbation of modular activities emerged in human brain specialization. In addition, the human-specific modules having neurobiological functions exhibited higher networking than other functional modules. This finding suggests that the expression of neural functions are more connected than other functions, and the resulting high-order brain functions could be identified as a result of consolidated inter-modular gene activities. Our result also showed that the functional module based transcriptome analysis has a potential to expand molecular understanding of high-order complex functions like cognitive abilities and brain disorders.

## Background

To characterize the molecular bases associated with human’s remarkably advanced high-order brain functions and vulnerability to various brain disorders, a comparative analysis between human and chimpanzee brain transcriptome is considered as an important way [1]. Until now, several previous studies compared the transcriptome data of human and chimpanzee brains. Despite some successes, little has been understood to account for unique features of human brain. In the previous study, we showed that a co-expression analysis of functional modules has shown increased sensitivity for identifying implication of more diverse genes and cellular functions that were previously undetected [2]. Recent studies have shown the existence of inter-module co-expression networks in the human brain, suggesting that systems-level relationships might also uniquely constitute human brain specificity [3, 4]. However, the implication of functional modules and their network properties have not been investigated in depth to further extract functional meanings of each module and their interplay with respect to human brain specialization. In this study, we analysed to find molecular characteristics of human brain functions using the human-specific co-expressed functional modules (HS-COMODs) and the chimpanzee-specific co-expressed functional modules (CS-COMODs). Our new approach using integrated analysis of gene expression data should be an aid in molecular interpretation of other complex biological functions too.

## Results

### Association of HS-COMODs and brain disorders

**Fig. 1:**
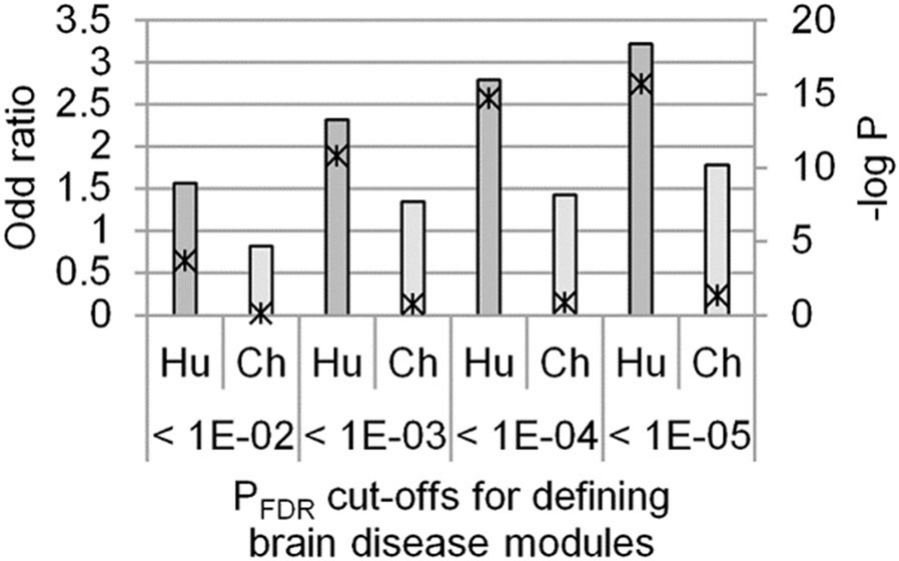
Overlap between species-specific modules and brain disease modules. The association between the species-specific co-expression and brain disease relevance of the functional modules was measured using the one-sided Fisher’s exact test

We questioned if the human vulnerability to neurodegenerative and psychiatric diseases might be relevant to the emergent co-expression of the functional modules in the human brain. To examine the possibility, we compared the HS-COMODs and the CS-COMODs with the brain disease modules. Functional modules associated to neuro/psychological diseases were defined using the Fisher’s exact test. The association between the species-specific co-expression and brain disease relevance of the functional modules was measured using the one-sided Fisher’s exact test. Our analysis showed that there was a statistically significant overlap between the HS-COMODs and the brain disease modules (Fig. 1). The association was observed more strongly with a more stringent cut-off used for defining the brain disease modules. On the other hand, such association was not observed for the CP-COMODs. This data suggests that the functional modules that were emergently functionalized in the human brain might comprise the cellular functions sensitive to the genetic perturbations and the etiology.

### Systems-level characteristics of HS-COMODs

**Fig. 2:**
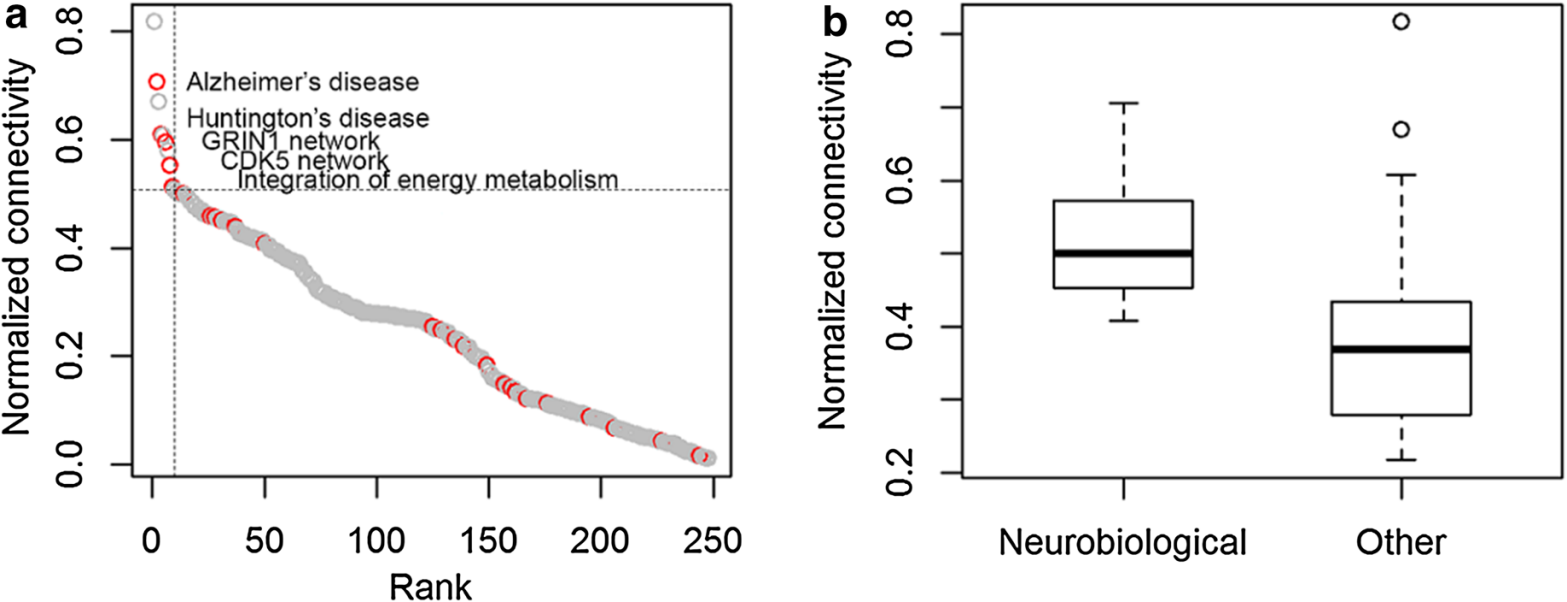
Normalized connectivity of HS-COMODs. Normalized connectivity of the HS-COMODs in the inter-module co-expression network. **a** The normalized connectivity of HS-COMODs. The *red circle* indicates the neurobiological modules. **b** The normalized connectivity of the neurobiological modules and that of the others

To extract the functional meanings, we analysed the inter-module co-expression network based on the network connectivity. Since the number of non-overlapping functional modules was different for each functional module, we normalized the connectivity by dividing by the number of non-overlapping HS-COMODs. The HS-COMODs ranked with the top 10 highest normalized connectivity (one-sided Fisher’s exact test, P = 1.52E−03) (Fig. 2a) includes pathways for ‘Alzheimer’s disease’, ‘Huntington’s disease’, ‘GRIN1 network’, ‘Integration of energy metabolism’, and protein interaction networks pivoted by GRIN1 and CDK5, all of which show implication to the learning and memory.

There are 25 neurobiological modules in total 248 non-overlapping HS-COMODs in human-specific functional module networks. In top 5 % (13 functional modules) HS-COMODs which have highest normalized connectivity, there are 5 neurobiological modules (one-sided Fisher’s exact test, P = 4.53E−03). In addition, average connectivity score of neurobiological modules is 0.308 compare to average connectivity of total functional modules and non-neurobiological module is 0.246 and 0.239, respectively. These results showed that the neurobiological modules had significantly higher normalized connectivity than did the other modules. In addition, the neurobiological modules had higher normalized connectivity than the rest, particularly in the ‘denser’ network (Fig. 2b). Our data suggests the central importance of the neurobiological modules, several of which are influential to the high-order brain functions such as learning or memory, and the prevalent functional cooperation of the neurobiological modules with a wide variety of functional modules in human brain.

We identified the HS-COMODs with the largest normalized connectivity to the neurobiological modules. More than half of the HS-COMODs with normalized connectivity over than 0.5 were involved in protein modification which was overrepresented functional category in our results (Table 1); especially, they were associated with protein ubiquitination. The concerted activity of the individual players such as the E3 ubiquitin ligase and proteasome upon the intra and extra-cellular stimuli might be crucial, as it has been shown that ubiquitin-dependent protein degradation delicately controls the life cycle of synaptic proteins for synapse regulation and organization, and in turn controls learning and memory [5, 6]. Implications of ubiquitin system have been also reported in previous analyses [7, 8] but their unique characteristics in terms of functional interplay have not been noted. Several E3s, such as AMFR, RNF5, and PARK2, involved in ‘protein ubiquitination’, were shown to specifically target the synaptic proteins involved in, for example, the postsynaptic density (GO:0014069) and nerve–nerve synaptic transmission (GO:0007270). The other modules, those that are highly connected to the neurobiological modules such as ‘HIV Infection’, and ‘generation of precursor metabolites and energy’ are also intriguing as it has been shown that positive manipulation of the energy metabolism may be effective in preventing or reversing cognitive impairments [9] and that the impaired immune function in HIV patients may lead to dementia, manifesting the cognitive dysfunction [10]. In summary, inter-module co-expression analysis among the HS-COMODs revealed functional interplay between specific and distinct functional modules. The neurobiological HS-COMODs tend to show co-expression with other diverse HS-COMODs, implying that the high-order human brain function such as cognition has been emerged by the systemic and parallel change of multiple functional modules in a concerted manner.

**Table 1:** Top 10 HS-COMODs showing highest connectivity to neurobiological modules

ID	Type	Name	Normalized connectivity
86	Biological process	Negative regulation of protein modification process	0.78
250	Pathway	HIV Infection	0.67
45	Biological process	Generation of precursor metabolites and energy	0.64
200	Hub network	RNF5 hub protein network	0.58
226	Pathway	Ubiquitin mediated proteolysis (KEGG)	0.56
284	Pathway	Protein ubiquitination (UniPathway)	0.56
120	Biological process	Protein ubiquitination	0.55
118	Biological process	Protein modification by small protein conjugation	0.55
38	Biological process	Energy derivation by oxidation of organic compounds	0.54
117	Biological process	Proteasomal ubiquitin-dependent protein catabolic process	0.53

## Discussion

It is important to unravel the molecular mechanisms underlying the high-order human brain functions for the drug development for the cognition enhancement. Despite tremendous researches, only few drugs have been approved [11] and the efficacy of those drugs for both healthy people and patients with cognitive dysfunctions seems to be modest and even harmful in some ways [12]. Recently it has been suggested that targeting multiple genes or complementary mechanisms by multiple drugs would be more desirable approach for the improved therapeutics [13, 14]. Moreover, it has been shown that evolutionarily emerging genes are likely to be the targets of successful drugs [15]. In these regards, this study might provide insight to the drug development. To make it more plausible, it would be further necessary to elucidate the specificity of the transcription factors or the epigenetic regulators on those key functional modules.

## Conclusion

We showed the significant overlap between the HS-COMODs and the brain disease modules, suggesting that the emergent functionalization of modular activities in human brain might be sensitive to the perturbations. In a system-level analysis into the HS-COMODs, it was shown that the functional modules show complex inter-module co-expression in human brain. Of particular note, the functional modules implicated to the neurobiological processes showed significantly higher connectivity than the others. From this point of view, neurobiological modules might have the potential contribution to cooperate with a wide variety of functional modules to drive cognitive functions at the systems level. Therefore, our findings showed that a systems approach adopted in the interpretation of transcriptomic change between human and chimpanzee brains has a potential to improve our molecular understanding of high-order complex functions like cognitive abilities and brain disorders.

## Methods

### Identification of brain disease modules

The genes associated with human brain disorders was compiled from public databases including the OMIM, the Genetic Association Database, the Cancer Gene Census, the KEGG Disease, and the HugeNavigator. The heterogeneous disease names were unified into a controlled vocabulary in the Unified Medical Language System (UMLS) using MetaMap. The individual diseases converted to the UMLS terms were further categorized into the 298 groups of higher classifications available in the International Classification of Diseases 10 provided by the World Health Organization. Among these 298 disease groups, we manually inspected and selected 15 categories (Table 2) as representative brain disease. To identify brain disease module, we used the one-sided Fisher’s exact test for the function enrichment analysis. The brain disease module was defined to be any functional module [16, 17] significantly enriched (FDR adjusted P <0.01) for the known genes associated with any brain disease category among the 15 selected categories.

**Table 2:** Disease categories used to compile brain disease modules

Disease categories
Alzheimer’s disease
Cerebral infarction
Cerebral palsy and other paralytic syndromes
Dementia
Epilepsy
Mental and behavioural disorders due to other psycho-active substance use
Mental and behavioural disorders due to use of alcohol
Mental retardation
Mood (affective) disorders
Nerve, nerve root and plexus disorders
Neurotic, stress-related and somatoform disorders
Other congenital malformations of the nervous system
Other diseases of the nervous system
Parkinson’s disease
Schizophrenia, schizotypal and delusional disorders

### Construction of co-expression network and characterization of inter-modular correlation

**Fig. 3:**
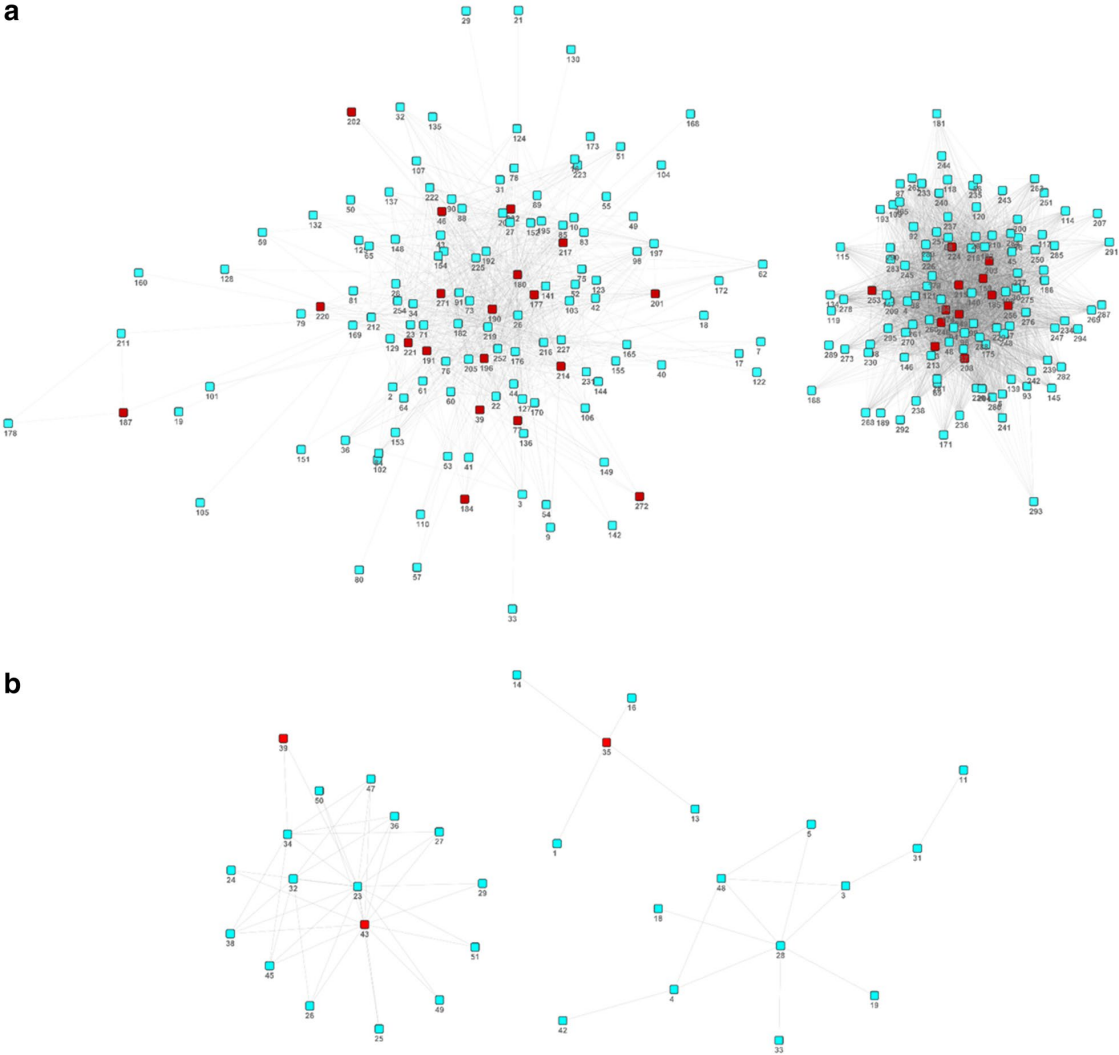
Co-expression network of HS-COMODs and CS-COMODs. **a** Co-expression network of HS-COMODs. **b** Co-expression network of CS-COMODs. The neurobiological modules were colored with *red*

We constructed the each species-specific functional modules networks. Each of the HS-COMODs and the CP-COMODs was represented by the module eigengene which correspond the first principal component extracted from the expression levels. We used the ModuleColor and WGCNA R packages to determine the module eigengene. Next, the co-expression between every pair of module eigengenes was calculated with the Pearson’s correlation coefficient and the significance of the co-expression was assigned to each pair among the 277 distinct HS-COMODs and the 49 distinct CP-COMODs, respectively [18]. The p values were adjusted using the Benjamini and Hochberg method. However, we restricted our analysis to pairs of each species-specific co-expressed functional modules with no overlapped component genes on the microarray data, since the overlapping genes significantly affect the co-expression measure. Using the significantly coexpressed pairs of HS-COMODs and CP-COMODs (FDR adjusted P <0.01), we produced the two human-specific functional module networks, one with higher density (Fig. 3a) and the three small and sparse chimpanzee-specific functional module networks (Fig. 3b).
